# Host Transcriptome and Microbiota Signatures Prior to Immunization Profile Vaccine Humoral Responsiveness

**DOI:** 10.3389/fimmu.2021.657162

**Published:** 2021-05-10

**Authors:** Elena Gonçalves, Yolanda Guillén, Javier R. Lama, Jorge Sanchez, Christian Brander, Roger Paredes, Behazine Combadière

**Affiliations:** ^1^ Sorbonne Université, INSERM, Centre d’Immunologie et des Maladies Infectieuses (CIMI-Paris), Paris, France; ^2^ IrsiCaixa AIDS Research Institute-HIVACAT, Hospital Universitari Germans Trias i Pujol, Barcelona, Spain; ^3^ Asociacion Civil Impacta Salud y Educacion, Lima, Peru; ^4^ Centro de Investigaciones Tecnológicas, Biomedicas y Medioambientales, Universidad Nacional Mayor de San Marcos, Lima, Peru; ^5^ Faculty of Medicine, Universitat de Vic-Central de Catalunya (UVic-UCC), Vic, Spain; ^6^ Institució Catalana de Recerca I Estudis Avançats (ICREA), Barcelona, Spain; ^7^ Infectious Diseases Department, Hospital Universitari Germans Trias, Barcelona, Spain

**Keywords:** biomarkers, vaccination, transcriptomic, microbiota, neutralizing antibodies, systems biology

## Abstract

The identification of new biomarkers is essential to predict responsiveness to vaccines. We investigated the whole-blood transcriptome and microbiome prior to immunization, in order to assess their involvement in induction of humoral responses two months later. We based our analyses on stool and skin microbiota, and blood transcriptome prior to immunization, in a randomized clinical study in which participants were vaccinated with the MVA-HIV clade B vaccine (MVA-B). We found that the levels of neutralizing antibody responses were correlated with abundance of *Eubacterium* in stool and *Prevotella* in skin. In addition, genus diversity and bacterial species abundance were also correlated with the expression of genes involved in B cell development prior to immunization and forecast strong responders to MVA-B. To our knowledge, this is the first study integrating host blood gene expression and microbiota that might open an avenue of research in this field and to optimize vaccination strategies and predict responsiveness to vaccines.

## Introduction

The largest fraction of immune cells is found at sites colonized by microorganisms, such as the skin or the gastrointestinal (GI) tract (1). The gut microbiome is essential for the development, maturation, and adequate functioning of the immune system (1, 2). The human skin is colonized during the postnatal period by microorganisms that prevent the invasion of external pathogens. Crosstalk between these commensals and the immune system is necessary to trigger innate and adaptive immune responses. Increased attention to the relation between the gut commensal bacteria and host immune responses has led scientists to question whether these microorganisms affect the efficacy of vaccines (3, 4). Moreover, although the composition of fecal microbiota may be one of the multiple factors that modulate host responses to external immunization, little is known about its role in the interindividual disparity in vaccine efficacy.

The first observation of a potential link between the microbiome and vaccines occurred in an oral vaccination model that used a heat-labile enterotoxin from *Escherichia coli* as an adjuvant (5, 6). In that situation, depletion of the intestinal microbiota was associated with a profound depression of antigen-specific Th1 and Th17 lymphocytes. Similarly, high antibody responses to the seasonal trivalent influenza vaccine (TIV) and polio vaccine (IPOL) require the presence of intestinal commensals (7). Inversely, impaired in microbiota composition and diversity have been reported to attenuate immune responses to vaccines (8). Interestingly, in human infants receiving hepatitis B, diphtheria, tetanus, and *Haemophilus influenza* type B vaccines, a randomized placebo-controlled double-blind trial demonstrated that vaccine-specific immune responses were enhanced by probiotics (9, 10). Recently described cross-reactivity between gut microbiota antigens and naive and memory CD4^+^ T and B cells (11) suggests that the antibody response to HIV-1 immunization may be shaped by intestinal B cells stimulated by host commensals (12). The microbiota is known to be required for a mature B-cell compartment (13). For example, germ-free mice have abnormalities in their B-cell systems and lower IgA levels than colonized animals of the same genotype (14). Microbial antigens and microbial metabolites, such as short-chain fatty acids, strongly promote plasma cell differentiation at mucosal and systemic sites (15). These microbial metabolites promote IgA production by regulating the metabolism and gene expression in B cells in mice models and in *in vitro* study of human B cells (15, 16). This IgA appears to orchestrate the beneficial mutualism established between the host and gut commensal microbiome by interacting directly with microbiota species.

Presentation of microbial antigens by the different MHC genotypes also contributes to modifying the IgA repertoires, which in turn modulate the composition of the microbiota in the gut (17). Accordingly, the depletion of anti-inflammatory microbial species and an expansion of proinflammatory species have been observed in human selective IgA deficiency (18). A lack of intestinal microbial stimulation results in fewer IgA^+^ plasma cells in the gut and a lower abundance of IgA in mouse models (19–21). Thus the diversity of IgA on the mammalian intestinal surface matches the intestinal taxa diversity (22). For these reasons, host microbial profiling during vaccine administration to might help optimize the vaccine responses and improve the tolerability of multiple antipathogen treatments. The microbiota, after all, constitutes a constant source of natural adjuvants capable of activating a multitude of pathways that control innate and adaptive immunity (23).

Systems biology has been successfully used to investigate the fundamental innate immune mechanisms orchestrating protective adaptive responses after the perturbation of vaccination against yellow fever (24, 25), HIV (26), Ebola (27), and influenza (28). An important challenge, however, is to analyze individual baseline human health characteristics to help identify those at higher risk of infection despite vaccination. Until now, only a few studies have looked for candidate traits associated with vaccine responsiveness and partially predicting the humoral response to vaccination against influenza (29–32). No study has examined the interrelations between each individual’s immunological state, their microbiota at baseline, and the impact of both on their vaccine-induced immune responses. As the most successful vaccines act through the production of antibodies (33), identifying specific individual characteristics at baseline should enhance our ability for dividing vaccines into “high responders” or “low responders” (34). Such predictive markers might serve as a potential diagnostic tool that assists vaccine development by taking into account the interindividual heterogeneity of immune responses.

This study used a systems biology approach to investigate the volunteers’ immune predisposition to respond to MVA-B vaccination, assessed by their blood transcriptome profile; specifically, that related to their B cell differentiation stages, and its conditioning by the human microbiota before vaccination. That is, we investigated the host gene expression in blood by a microarray approach and the skin and stool microbiota by using 16S ribosomal RNA sequencing both before vaccination. The objective was to examine their potential involvement in an effective MVA-B neutralizing antibody (Nabs) response during the CUTHIVAC 03 randomized phase Ib clinical study. As published in a previous work (35), CUTHIVAC 03 clinical study included 10 HIV seronegative subjects aged from 18 to 45 years by the intramuscular route with MVA-HIV clade B vaccine. We analyzed their baseline transcriptomic signature and baseline bacterial species abundance and diversity in skin and stool to assess their potential association with the intensity of the Nabs response.

## Materials and Methods

### Clinical Study

The CUTHIVAC-003 phase Ib randomized clinical study enrolled 20 volunteers aged 18–45 years at low risk of HIV infection, from October 15, 2014, to November 19, 2015 (35). The participants received HIV-1 MVA Clade B vaccine by either t.c. or i.m. administration (1:1 ratio) after randomization for allocation at the Clinical trial unit (CTU) of the Asociacion Civil Impacta Salud y Educacion (IMPACTA) in Peru (35). The volunteers received vaccine on week (w) 0 by the allocated route of administration to assess its safety and immunogenicity against the MVA vector alone and against MVA-B. In this paper we focused our analyses on the 10 volunteers vaccinated by i.m. route and the MVA-specific neutralizing antibody responses. The MVA-B vaccine encodes a multi-HIV antigen, specifically a synthetic fusion protein comprising nearly complete protein sequences from the Gag, Pol, and Nef genes of the HIV-1 IIIB strain and the nearly complete protein encoding sequence from the Env gene obtained from the HIV BX08 strain (36). The group received 1 x 1.0 mL of the MVA-B preparation at 1 x 10^8^ PFUs (Plaque-Forming Units) by needle injection to the muscle of the deltoid region of the nondominant arm. The MVA-HIV Clade B vaccine has been used in several clinical studies (36–39), and CUTHIVAC-003 was the fifth human experiment with this MVA vector expressing HIV-B antigens. The amplitude of the humoral response was assessed by measuring neutralizing antibodies specific to the MVA vector in serum (NAb). Exploratory analyses used whole blood to study baseline gene expression.

### Ethics and Community Involvement

The study was conducted in accordance with the Declaration of Helsinki and the International Conference on Harmonization Good Clinical Practice guidelines and approved by the relevant regulatory and independent ethics committees. Each participant provided written informed consent before study entry. The study was registered and approved by the Peru regulatory authorities (IMPACTA IRB 0037-2014-CE; Peru NIH 396-2014-OG-OGITT-OPE/INS).

### Skin and Feces Sampling

For each individual, skin swab samples from the deltoid muscle region (~5-20 cm below the vaccine administration site) were collected before the vaccination (w0). Skin samples were collected with Catch-All™ Sample Collection Swab kits moistened with SCF-1 solution. The skin surface was sampled for 30 seconds by firmly swabbing the cotton tip back and forth ~50 times. The cotton tip was stored in sterile tubes with MoBio solution at -80°C until DNA extraction. Fecal samples for each participant were collected in sterile fecal collection tubes the day before the vaccination, matching the skin sample time points. All samples were stored at 4–5°C until their reception at the IMPACTA clinical trial site, where they were cryopreserved at -80°C. All samples were shipped on dry ice to the IrsiCaixa AIDS Research Institute for DNA extraction, amplification, and sequencing.

### DNA Extraction and Amplicon Sequencing From Skin and Fecal Samples

DNA extraction was performed with the DNA Extraction kit from Epicentre Technologies© (Madison, WI, USA). Six aliquots of buffer solution from the DNA extraction kit were used as negative controls. To amplify the variable V3-V4 region from the 16S rRNA gene, we used the primer pair described in the MiSeq™ rRNA Amplicon Sequencing protocol, which already has the Illumina adapter overhang nucleotide sequences added to the 16S rRNA V3-V4-specific primers, i.e., 16S_F 5’-(TCG GCA GCG TCA GAT GTG TAT AAG AGA CAG CCT ACG GGN GGC WGC AG)-3’ and 16S_R 5’-(GTC TCG TGG GCT CGG AGA TGT GTA TAA GAG ACA GGA CTA CHV GGG TAT CTA ATC C) -3’. Amplifications were performed in triplicate 25-μL reactions, each containing 2.5 μL of non-diluted DNA template, 12.5 μL of KAPA HiFi HotStart Ready Mix (containing KAPA HiFi HotStart DNA Polymerase, buffer, MgCl2, and dNTPs, KAPA Biosystems Inc., Wilmington, MA, USA), and 5 μL of each primer at 1 μM. Thermal cycling conditions consisted of an initial denaturation step (3 minutes at 95°C), followed by 30 cycles of denaturation (30 seconds at 95°C), annealing (30 seconds at 55°C), and extension (30 seconds at 72°C). These were followed by a final extension step of 10 minutes at 72°C. Once the desired amplicon was confirmed in 1% agarose gel electrophoresis, all three replicates were pooled and stored at -30°C until the sequencing library was prepared. After amplified DNA templates were cleaned up for non-DNA molecules and Illumina sequencing adapters and dual indices attached with the Nextera XT Index Kit (Illumina, Inc., San Diego, USA), the corresponding PCR amplification program was run, as described in the MiSeq 16S rRNA Amplicon Sequencing protocol. After a second round of cleanup, amplicons were quantified with the Quant-iT™ PicoGreen^®^ dsDNA Assay Kit (Invitrogen, Carlsbad, MA, USA) and diluted in equimolar concentrations (4 nM) for further pooling. Sequencing was performed on an Illumina MiSeq™ platform (Illumina, Inc., San Diego, USA) according to the manufacturer’s specifications to generate a median of 30,644 paired-end sequences of ~300 bp length in each direction (~61,289 reads per sample).

### Sequence Quality Control and Microbiota Analysis

The quality of MiSeq raw sequences was assessed with the FastQC software (40) (http://www.bioinformatics.babraham.ac.uk/projects/fastqc/). Sequences were trimmed with Trimmomatic (41), with a cutoff value of Q30 for both ends, a minimum mean threshold of Q20 for 30-bp-sliding window across sequences, and a minimum read length of 250 bp (**Supplemental Figures 1A, B**). After quality control, 28 samples including controls (n = 8) and volunteers (n = 10, 5 women and 5 men) for skin and stools, were further analyzed. Mothur pipeline (42) was used to bin 16S rDNA sequences into operational taxonomic units (OTUs) with a threshold of 97% sequence similarity. OTUs present in only a single sample were discarded. Rarefaction curves were represented by defining the maximum subsampling size as the number of sequences of the sample with the fewest sequences (2751 sequences for skin samples, and 1059 sequences for stool samples) (**Supplemental Figure 1C**). Richness and diversity indexes were estimated by using the summary. Single module implemented in mother. For taxonomical analysis, 16S rDNA sequences were classified according to the GreenGenes database (43) version 13.5.99.

### MVA-GFP Neutralizing Antibody Assay

Anti-MVA neutralizing activities were evaluated in serum collected at week 8 (w8) with an assay based on GFP detection by flow cytometry (44, 45). It used HeLa cells as targets and a recombinant strain of MVA expressing the enhanced Aequoriae GFP (36). Serial 2-fold dilutions of heat inactivated serum were performed in 96-well round-bottom tissue culture plates (TPP, Zurich, Switzerland) containing DMEM (Gibco, Invitrogen, Waltham, Massachusetts, USA) supplemented with 2% fetal calf serum (PAA, Laboratories GmbH, Pashing, Austria). MVAeGFP was then added to each well at a MOI of 0.25. The plate was then incubated for 1 hour at 37°C until the addition of 1 × 10^5^ HeLa cells. The incubation then continued for an additional 16 hours at 37°C, 0.5% CO_2_. After trypsinization, the cells were washed with PBS supplemented with 0.5% fetal calf serum and 2 mM EDTA and fixed with 2% formaldehyde. GFP expression was analyzed with FACSCanto II and Diva software (BD Biosciences, San Jose, CA, USA). The percentage of neutralization was defined as the ratio of the reduction in the number of GFP-expressing cells to the number of GFP-expressing cells in untreated control wells.

### RNA Extraction and Data Preprocessing for Transcriptomic Analysis

Whole blood samples of 2.5 mL were collected in PAXgene RNA tubes (PreAnalytix) twice from each volunteer two weeks before (w-2) and the day of the vaccination (w0). These tubes enable the preservation and stabilization of RNA (storage at -80°C). Total RNA was extracted from whole blood according to the instructions in the handbook accompanying the PAXgene blood RNA Kit (PreAnalytiX, Hombrechtikon, Switzerland). RNA purity and integrity were assessed on the Agilent 2100 Bioanalyzer with the RNA 6000 Nano LabChip reagent set (Agilent, Palo Alto, CA, USA). Samples for microarray hybridization were prepared as described in the Affymetrix GeneChip WT PLUS Reagent Kit User Manual (Affymetrix, Inc., Santa Clara, CA, USA). For hybridization (to Affymetrix Human Gene 2.1 ST Array Plates), washing, staining, and scanning took place in an Affymetrix GeneTitan system, controlled by the Affymetrix GeneChip Command Console software w4.2. Background signal correction was performed by applying the background. Correct function from the limma package on the perfect match (PM) signals with R Software 3.3.1. The underlying model is the normal-exponential convolution model from RMA (chip intensity: addition of a signal exponentially distributed, chip noise: follows Gaussian distribution) (46). The variance stabilizing transformation algorithm (justvsn function from the vsn package (47) was applied to the background corrected signal (monotonic transformation), and the signal then transformed back to its usual scale by exponentiation (base 2). To make the chips comparable, a quantile normalization (48) (normalize function from the affy package) was then applied to the variance-stabilized signal. The probe signals for replicated arrays were averaged and a quantile normalization performed anew (Altrabio, Lyon, France). In all, 24,768 probes were analyzed.

### Statistical Analysis

Microbiome samples were clustered according to their genus composition by a nonmetric multidimensional scaling (NMDS) approach based on ecological distance matrices calculated by Bray-Curtis dissimilarities, as implemented in R (Vegan, metaMDS, and ggplot2 packages). NMDS ellipses were drawn based on a confidence interval (CI) of 0.95. To determine significant factors that describe the community structure better, we used a multivariate ADONIS test with terms added sequentially. The associations between baseline genus abundance or genus diversity, blood gene expression, and MVA-Nab response were evaluated by using the Spearman rank correlation test with significance defined by a *P*-value <0.05. The heatmap was performed with values row-centered and scaled, Pearson correlation as the distance method and a dendrogram computed and reordered based on row means. The heatmap, logistic regression analyses, and ROC curves were performed and generated with R. Ingenuity^®^ pathway analysis (IPA, Qiagen, Redwood City, CA, USA) was used to perform functional enrichment analyses and identify new targets or candidate biomarkers within the context of biological systems. It provided the canonical pathways, molecular/cellular functions, and networks that were statistically overrepresented in the gene signatures.

### Sequence and Data Availability

The normalized microarray data that support the finding of this study have been deposited in ArrayExpress with the accession code E-MTAB-9642. Raw Illumina MiSeq sequences and study metadata were deposited in the National Center for Biotechnology Information - NCBI repository (Bioproject accession: PRJNA691892, Samples accessions: SAMN17307480 to SAMN17307549). The sequence of the identified biomarkers may be found with the NCBI Reference, for IGLV8-61: NG_000002 and NC_000022 (Reference GRCh38.p13 Primary Assembly); for BLK: NM_001715/NM_001330465 (isoform 1 and isoform 2) and NP_001317394/NP_001706 (isoform 1 and isoform 2) and for EBF1: NM_001290360 and NP_001277289 (isoform 1)/NM_024007 and NP_076870 (isoform 2)/NM_182708 and NP_874367 (isoform 3)/NM_001324101 and NP_001311030 (isoform 4)/NM_001324103 and NP_001311032 (isoform 5)/NM_001324106 and NP_001311035 (isoform 6)/NM_001324107 and NP_001311036 ((isoform 7)/NM_001324108 and NP_001311037 (isoform 8)/NM_001324109 and NP_001311038 (isoform 9)/NM_001324111 and NP_001311040 (isoform 10)/NM_001364155 and NP_001351084 (isoform 11)/NM_001364156 and NP_001351085 (isoform 13)/NM_001364157 and NP_001351086 (isoform 14)/NM_001364158 and NP_001351087 (isoform 15)/10 NM_001364159 and NP_001351088 (isoform 16).

## Results

### Study of Host Microbiota Before Vaccination and Relation to Post-Vaccination Humoral Responses

**Figure 1:**
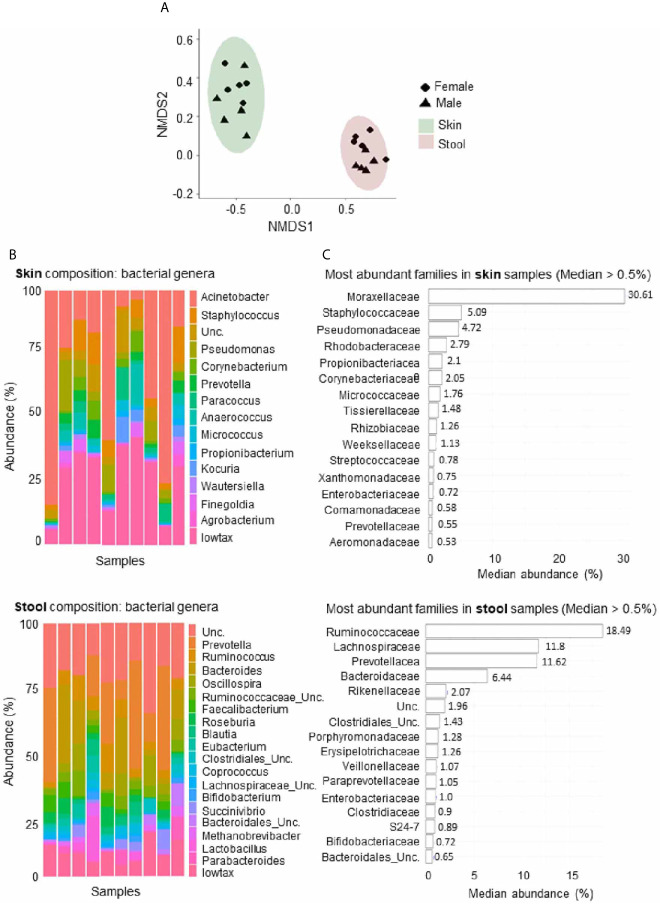
Microbial composition of fecal and skin samples. **(A)** Nonmetric multidimensional scaling plot based on Bray-Curtis microbiome distances showing different configurations between skin and stool samples. Ellipses represent 95% confidence intervals. **(B)** Abundance of genera with a median abundance >5% across skin (upper part) and stool (lower part) samples. The lowtax taxa corresponds to the collection of those genera that have a median abundance <0.5%. Unc, unclassified. **(C)** Microbial families with a median abundance >5% across skin (upper part) and stool (lower part) samples are ranked according to their median abundance.

The study included five men and five women (18-45 years old) vaccinated by the intramuscular route to assess the safety and immunogenicity of MVA-HIV clade B (MVA-B), results reported elsewhere (35). Exploratory analysis of whole blood samples at two distinct time-points before vaccination (w-2 and w0) studied the gene expression profile and the skin and stool samples for microbiome analysis (w0) at baseline. As expected, the microbial composition differed between the skin and stool samples (**Figures 1A–C**). In addition, the stool samples showed dissimilarities between men and women, but this comparison did not reach statistically significant differences (*P* < 0.097) (**Figure 1A**). The predominant microbial families relatively abundant in skin samples were *Moraxellaceae*, *Staphylococcaceae* and *Pseudomonadaceae*, whereas *Ruminococcaceae*, *Lachnospiraceae*, *Prevotellaceae*, and *Bacteroidaceae* were predominant in stool samples (**Figures 1B, C**). The 16S RNA sequencing generated several metrics: richness (sobs: number of observed OTUs; chao: Chao1 richness estimate; ace: Abundance-based coverage estimation) and diversity (Shannon: Shannon diversity index; sd_invsimpson: inverse Simpson diversity index).

**Figure 2:**
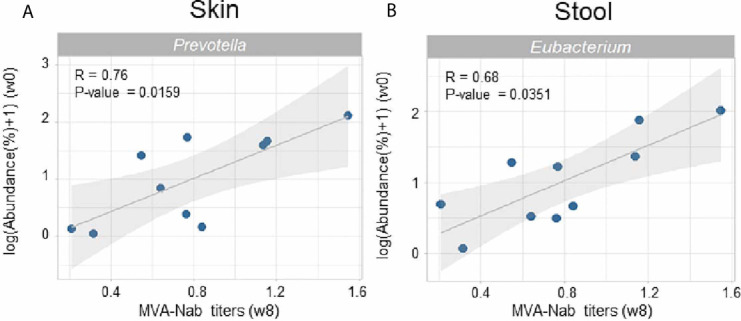
Microbial abundance before vaccination is correlated with MVA-Nab responses. **(A)** Abundance of *Prevotella* in skin and **(B)** abundance of *Eubacterium* in stool are correlated with MVA-specific neutralizing antibody titers at w8 (log(EC50)). Spearman rank sum test was applied with a *P*-value < 0.05. All genera were filtered by a minimum median abundance of 0.1% across the samples.

The amplitude of the humoral response was defined by the MVA-specific IgG neutralizing antibodies measured in serum at w8 post-vaccination (35). We observed no correlation between the MVA-Nab response and the baseline indexes of diversity and richness in either skin or stool (data not shown). We did however find significant positive correlations between the abundance of both skin *Prevotella* (r = 0.76, *P* = 0.0159) (**Figure 2A**) and fecal *Eubacterium* (r = 0.68, *P* = 0.0351) (**Figure 2B**) at baseline with MVA-Nab response.

### Whole Blood Gene Expression and Host Microbiota Before Vaccination Are Associated With Post-Vaccination Humoral Responses

**Figure 3:**
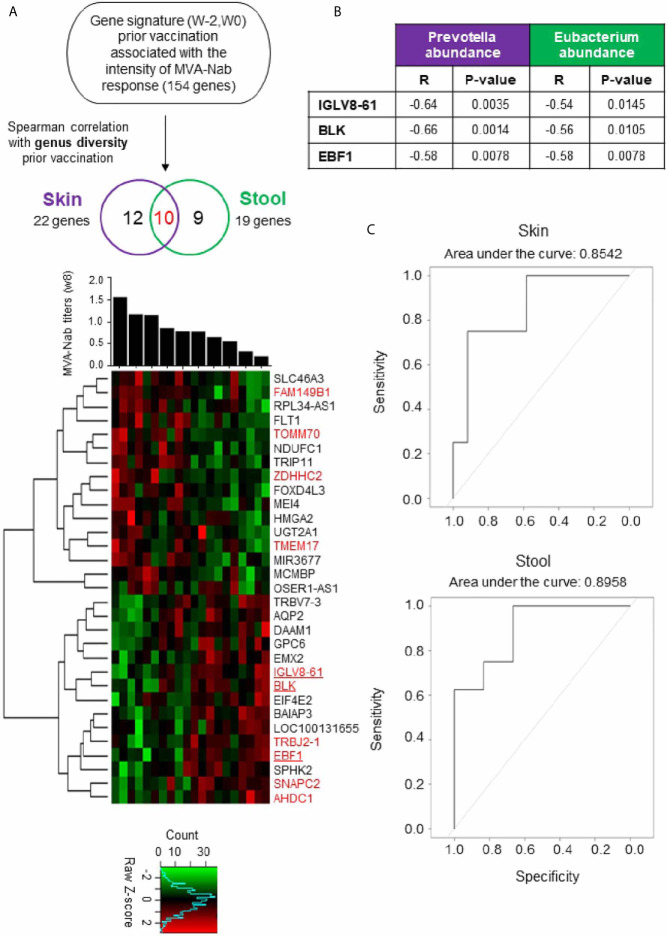
Blood gene expression combined with host microbiota before vaccination shapes MVA-B responses **(A)** Investigation of the blood gene expression (w-2, w0) correlated with MVA-Nab response (w8) and host genus diversity (w0), for skin (purple) and stool (green). The Spearman correlation test was applied with an adjusted *P* < 0.05 (r < -0.6 and r > 0.6) defined as statistically significant. The heatmap shows the expression profile of the 22 (purple) and 19 (green) genes correlated respectively with skin and stool, according to MVA-Nab response intensity from highest to lowest responders. The 10 common genes are colored in red. The color-gradient from green (-2, low) to red (2, high) indicates the intensity of gene expression. Among the genes correlated with both skin and stool, three genes are underlined because they are involved in B cell functions. **(B)** Table shows the significant correlation coefficients and *P*-values for each of the three genes with the abundance of the genus correlated with MVA-Nab response. **(C)** ROC curves show the specificity and the sensitivity of the logistic regression models, i.e., the proportion of correctly predicted responders and nonresponders, respectively. The logistic regression is based on the expression of the minimal gene signature (IGLV8, EBF1, and BLK) and the abundance of *Eubacterium* and *Prevotella*, respectively, in stool and skin.

To improve our understanding of host molecular mechanisms potentially associated with skin and gut microbiota that may be involved in vaccine immunogenicity, we counted the number of genes at baselines that were correlated with the MVA-Nab response at w8. We confirmed that gene expression of the baseline samples did not differ between w-2 and w0 using hierarchal clustering analysis (data not shown). Out of all samples, we found 154 significant genes correlated with the MVA-Nab response (adjusted P < 0.05; r < -0.6 and r > 0.6) (**Figure 3A**). However, no correlation was observed between genus diversity and MVA-Nab response.

Next, we looked for a correlation between the microbiota diversity index and the genes (n = 154) correlated at baseline with MVA-Nab responses. We found 22 genes for skin and 19 for stool that were correlated with at least one diversity index (Shannon or sd_invsimpson) (**Figure 3A** and **Tables 1**, **2**), including 10 common genes to the skin and stool samples. Among these genes, we observed one gene cluster positively correlated with MVA-Nab response and another negatively correlated with humoral response (**Figure 2A**). According to the IPA analysis, the negatively correlated genes appear to be involved in protein transmembrane transport, translation and transcription regulation, cell division, migration, proliferation, and differentiation, as well as in the oxidation reduction and metabolic processes (**Table 2**). The positively correlated genes, on the other hand, appeared involved in cell homeostasis and migration, cell growth, proliferation, regulation of gene expression, the apoptotic process, exocytosis, and intracellular signal transduction (**Table 2**).

**Table 1:** Skin and stool genus diversity correlations with blood gene expression.

STOOL	Genus diversity sd_invsimpson		Genus diversity shannon	
Genes	r*	P-value	r*	*P*-value
AHDC1	-0.47	0.0361	*ns*	*ns*
**BLK**	**-0.66**	**0.0015**	**-0.64**	**0.0024**
**EBF1**	**-0.46**	**0.0418**	***ns***	***ns***
EIF4E2	-0.57	0.0090	-0.54	0.0145
EMX2	-0.48	0.0322	*ns*	*ns*
FAM149B1	0.52	0.0182	0.47	0.0375
FLT1	0.49	0.0264	0.44	0.0500
FOXD4L3	0.52	0.0181	0.47	0.0361
**IGLV8-61**	**-0.85**	**2.7116 e-06**	**-0.81**	**1.3721 e-05**
MEI4	0.48	0.0335	*ns*	*ns*
MIR3677	0.47	0.0375	*ns*	*ns*
NDUFC1	0.44	0.0500	*ns*	*ns*
OSER1	*ns*	*ns*	-0.44	0.0500
SNAPC2	-0.63	0.0027	-0.56	0.0100
TMEM17	0.56	0.0105	0.48	0.0322
TOMM70	0.61	0.0043	0.52	0.0182
TRBJ2	-0.45	0.0466	*ns*	*ns*
TRIP11	0.46	0.0434	*ns*	*ns*
ZDHHC2	0.50	0.0254	0.45	0.0450
**SKIN**	**Genus diversity sd_invsimpson**		**Genus diversity shannon**	
**Genes**	r*	**P-value**	r*	***P*-value**
AHDC1	-0.46	0.0389	-0.55	0.0121
AQP2	-0.4679	0.0375	-0.52	0.0174
BAIAP3	-0.48	0.0335	-0.46	0.0403
**BLK**	**-0.67**	**0.0011**	**-0.60**	**0.0048**
DAAM1	*ns*	*ns*	-0.5102	0.0215
**EBF1**	**-0.55**	**0.0127**	**-0.53**	**0.0166**
FAM149B1	*ns*	*ns*	0.46	0.0418
GPC6	*ns*	*ns*	-0.50	0.0254
HMGA2	0.47	0.0375	0.59	0.0067
**IGLV8-61**	**-0.56**	**0.0095**	**-0.54**	**0.0133**
LOC1001311655	-0.47	0.0348	*ns*	*ns*
MCMBP	0.49	0.0264	0.4679	0.0375
RPL34	0.51	0.0225	0.58	0.0078
SLC46A3	0.50	0.0234	0.5253	0.0174
SNAPC2	-0.49	0.0275	-0.53	0.0152
SPHK2	*ns*	*ns*	-0.51	0.0215
TMEM17	0.60	0.0051	0.68	0.0009
TOMM70	0.50	0.0234	0.57	0.0082
TRBJ2	-0.49	0.0286	*ns*	*ns*
TRBV7	*ns*	*ns*	-0.44	0.0500
UGT2A1	0.50	0.0244	0.55	0.0121
ZDHHC2	*ns*	*ns*	0.48	0.0310

*Correlation coefficient and P-value for the relations between genus diversity (shannon: diversity shannon index, sd_invsimpson: inverss simpson diversity index) and the genes correlated with MVA-Nab response in skin and stool conditions. Three genes involved in B cell function and correlated in both skin and stool are highlighted in bold. ns, not significant.

**Table 2:** Description of the genes from the minimal signatures for skin and stool conditions.

Symbol	Entrez Gene Name	Molecular Function	Canonical Pathway	Description	Biological process
**AHDC1**	AT-hook DNA binding motif containing 1	DNA binding	_	Gene mutations: Xia-Gibbs syndrome	_
**AQP2**	Aquaporin 2	Actin binding; channel activity; glycerol transmembrane transporter activity	Apelin endothelial signaling pathway; eNOS signaling	Water chanel protein from kidney collecting tubule.	Actin filament depolymerization; apoptotic process; excretion; cell homeostatis; glycerol transport
**BAIAP3**	BAI1 associated protein 3	Calcium ion binding; phospholipid binding; protein binding; syntaxin binding	_	P53-target gene encodes brain-specific angiogenesis inhibitor. Two C2 domains from proteins involved in signal transduction or membrane trafficking	exocytosis; G-protein coupled receptor signaling pathway; regulation of synaptic transmission; retrograde transport
**BLK**	**BLK proto-oncogene, Src family tyrosine kinase**	**ATP-binding; kinase activity; protein binding; transferase activity**	**PI3K Signaling in B lymphocytes; Tec kinase signaling**	**Involved in b cell proliferation and differentiation and has a role in B-cell receptor signaling and B-cell development**	**B cell receptor signaling pathway; cell differentiation and proliferation; innate immune response**
**DAAM1**	Dishevelled associated activator of morphogenesis 1	Actin binding; identical protein binding; protein binding; Rho GTPase binding	PCP pathway; Role of macrophages; Fibroblasts and Endothelial Cells	Involved in cell motility, adhesion, cytokinesis, reorganization of the actin cytoskeleton, cell polarity and movement	Actin cytoskeleton organization; Wnt receptor signaling pathway
**EBF1**	**Early B cell factor 1**	**RNA polymerase II core promoter proximal region sequence-specific DNA binding; transcription factor involved in positive regulation of transcription activity; DNA binding**	**B cell receptor signaling; IL-17 Signaling pathway**	**Transcriptional activator which recognizes variations of the palindromic sequence 5-ATTCCCNNGGAATT-3. Named Transcription factor COE1 and activates target genes**	**Multicellular organismal development; positive regulation of transcription; DNA-dependent; positive regulation of transcription from RNA polymerase II promoter**
**EIF4E2**	Eukaryotic translation initiation factor 4E family member 2	RNA binding; translation initiation factor activity; ubiquitin protein ligase binding	_	Recognizes and binds the 7-methylguanosine-containing mRNA cap during an early step in the initiation. Acts as a repressor of translation initiation	Negative regulation of translation
**EMX2**	Empty spiracles homeobox 2	Sequence-specific DNA binding RNA polymerase II transcription factor activity	_	Known expressed in three human tissues: dorsal telencephalon, olfactory neuroepithelium, and epithelial urogenital system	Brain development; Neuron differentiation; regulation of gene expression
**FAM149B1**	Family with sequence similarity 149 member B1	_	_	_	_
**FLT1**	Fms related tyrosine kinase 1	ATP binding; vascular endothelial growth factor-activated receptor activity; kinase activity; transferase activity; protein binding; transmembrane signaling receptor activity	eNOS signaling; IL-8 signaling; NF-kB signaling; STAT3 pathway	Binds to VEGFR-1, VEGFR-B and placental growth factor and plays an important role in angiogenesis and vasculogenesis. Expression of this receptor is found in vascular endothelial cells and peripheral blood monocytes	Cell differentiation; cell migration; cell proliferation; factor stimulus; monocyte chemotaxis;
**FOXD4L3**	Forhead box D4 like 6	protein binding; sequence-specific DNA binding; sequence-sequence DNA binding RNA polymerase II transcription factor activity	_	_	anatomical structure morphogenesis; cell differentiation; regulation of transcription from RNA polymerase II promoter
**GPC6**	Glypican 6	heparin sulfate proteoglycan binding; protein binding	_	The glypicans comprise a family of glycosylphosphatidylinositol-anchored heparin sulfate proteoglycans, are implicated in the control of cell growth and cell division. Putative cell surface coreceptor for growth factors, extracellular matrix proteins, proteases and anti-proteases.	cell migration; glycosaminoglycan biosynthetic process; regulation of signal transduction; retinoid metabolic process; Wnt receptor signaling pathway
**HMGA2**	High mobility group AT-hook 2	5’-deoxyribose-5-phosphate lyase activity; AT DNA binding; C2H2 zinc finger domain binding; cAMP response element binding; DNA-dependent protein kinase activity; MH1 domain binding; transcription regulation	Regulation of the Epithelial-Mesenchymal Transition Pathway	Belongs to the non-histone chromosomal high mobility group (HMG) protein family, as architectural factors and are essential components of the enhancesome.	Cell division; chromatin organization; DNA damage response; negative regulation of retroviral genome replication; negative regulation of apoptotic process
**IGLV8-61**	**Immunoglobulin lambda variable 8-61**	**Antigen binding**	**_**	**Participates in the antigen recognition. Membrane-bound antibodies or secreted glycoproteins produced by B lymphocytes. Trigger the clonal expansion and differentiation of B lymphocytes into immunoglobulins-secreting plasma cells.**	**adaptive immune response; immunoglobulin production**
**LOC100131655**	Uncharacterized	_	_	_	_
**MCMBP**	Mini-chromosome maintenance complex binding protein	chromatin binding; protein binding	_	Encodes a protein which is a component of the hexameric minichromosome maintenance (MCM) complex which regulates initiation and elongation of DNA	cell cycle; cell division; DNA-dependent DNA replication
**MEI4**	Meiotic double-stranded break formation protein 4	Protein binding	_	Required for DNA double-strand breaks formation in unsynapsed regions during meiotic recombination	DNA recombination; meiotic cell cycle; meiotic DNA double-strand break formation, synapsis
**MIR3677**	microRNA 3677	_	_	Non-coding RNAs involved in post-transcriptional regulation of gene expression in multicellular organisms by affecting both the stability and translation of mRNAs	_
**NDUFC1**	NADH: ubiquinone oxidoreductase subunit C1	NADH dehydrogenase (ubiquinone) activity	_	Subunit of the NADH: ubiquinone oxidoreductase, the first enzyme complex in the electron transport chain located in the inner mitochondrial membrane	Mitochondrial electron transport, NADH to ubiquinone; mitochondrial respiratory chain complex I assembly; oxidation-reduction process
**OSER1-AS1**	Oxidative stress responsive serine rich 1	_	_	_	Cellular response to hydrogen peroxide
**RPL34-AS1**	Ribosomal protein L34	Cadherin binding; RNA binding; structural constituent of ribosome	EIF2 Signaling	Component of the 60S subunit belongs to the L34E family of ribosomal proteins. It is located in the cytoplasm and overexpression of this gene has been observed in some cancer cells	Nuclear-transcribed mRNA catabolic process; nonsense-mediated decay; SRP-dependent cotranslational protein targeting to membrane; translation initiation
**SLC46A3**	Solute carrier family	_	_	Transmembrane protein, transports small molecules across membrane. Found in lysosomal membranes where it transports catabolites from the lysosomes to the cytoplasm. Effective transporter of the cytotoxic drug maytansine	Transmembrane transport
**SNAPC2**	Small nuclear RNA activating complex polypeptide 2	sequence-specific DNA binding transcription factor activity	_	Subunit of the snRNA-activating protein complex associated with the TATA box-binding protein. Is necessary for RNA polymerase II and III dependent small-nuclear RNA gene transcription	regulation of transcription; DNA-dependent; transcription from RNA polymerase III promoter
**SPHK2**	Sphinganine kinase	ATP binding; sphinganine kinase activity; nucleotide binding; Ras GTPase binding; sphingosine-1-phosphate receptor activity; transferase activity	Ceramide Signaling; PDGF Signaling	One of two sphingosine kinase isozymes, catalyses the sphingosine phosphorylation into sphingosine 1-phosphate. It mediates many cellular processes including migration, proliferation and apoptosis	cell proliferation; lipid phosphorylation; metabolic process; negative regulation of apoptotic process; positive regulation of cell proliferation; sphingosine metabolic process
**TMEM17**	Transmembrane protein 17	protein binding	_	Transmembrane component of the tectonic-like complex localized at the transition zone of primary cilia and acting as a barrier that prevents diffusion of transmembrane proteins between the cilia and plasma membrane. Required for ciliogenesis and sonic hedgehog/SHH signaling	Cell projection organization; cilium morphogenesis; smoothened signaling pathway
**TOMM70**	Translocase of outer mitochondrial membrane 70	protein binding; protein transmembrane transporter activity	Sirtuin Signaling Pathway	Import receptor of the outer mitochondrial membrane that is part of the translocase of the outer membrane complex. Involved in the import of mitochondrial precursor proteins	Negative regulation of cell growth involved in cardiac muscle cell development; protein targeting to mitochondrion; protein transmembrane transport; response to thyroxine stimulus
**TRBJ2-1**	T cell receptor beta joining 2-1	_	_	_	_
**TRBV7-3**	T cell receptor beta variable 7-3	_	_	_	_
**TRIP11**	Thyroid hormone receptor interactor 11	protein binding; transcription coactivator activity	Aryl Hydrocarbon receptor signaling	Interaction with thyroid hormone receptor beta. Associated protein with Golgi apparatus. Protein N-terminal region binds Golgi membranes and C-terminal binds the minus ends of microtubules; thus, the protein is thought to play a role in assembly and maintenance of the Golgi ribbon structure around the centrosome	Bone development; Golgi organization; intraflagellar transport involved in cilium morphogenesis; protein glycosylation; transcription from RNA polymerase II promoter; ventricular septum development

IPA software and Uniprot opensource were used to highlight molecular function, canonical pathway, description and biological process where are involved each gene of the minimal signature. The three genes signature involved in B cell function and correlated with MVA-Nab titers and baseline diversity and abundance of bacteria are highlighted in bold.

Interestingly, among the 10 common genes to the skin and stool samples we found the IGLV8-61, BLK, and EBF1 genes which are involved in antigen recognition, B cell development, proliferation, and differentiation, and in the positive regulation of transcription in B cell and B cell receptor signaling (**Figure 3A**). Surprisingly these three significant genes involved in B cell development stages were negatively correlated with the baseline abundance of *Prevotella* and *Eubacterium*, respectively for skin and stool (**Figure 3B**). To assess the predictive power of this signature of three genes and each of the two microbial genera, we ran logistic regression models (**Figure 3C**). Use of the expression of the three genes and *Prevotella* abundance in the skin microbiota has an 85.42% chance, assessed by its area under the curve, of correctly predicting MVA-Nab responders, while with the three-gene signature and *Eubacterium* abundance in the stool microbiota there is an 89.58% chance of correctly predicting MVA-Nab responders (**Figure 3C**). These results suggest that advanced B lymphocyte differentiation before vaccination, potentially signaled by high expression of these three genes, and associated with low abundance of *Prevotella* or *Eubacterium*, is associated with poor MVA-Nab response.

## Discussion

To our knowledge, this work is the first to investigate the potential relationship between pre-vaccination host gene expression in blood cells, skin and stool microbiota and their association with the intensity of ensuing post-vaccination Nab responses. The data may provide important guidance for future design and refinement of vaccine strategies aiming at the induction of neutralizing antibody-mediated immunity. The limitation of this study is the small number of individuals included. However, the strength of our work is the availability of two sets of gene expression data collected at baseline (w-2, w0) that is often absent in other studies. It is intriguing to discover three genes, all involved in B cell differentiation and proliferation correlated with humoral responses 2 months later. Further validation studies are necessary in the future.

First, we observed that the abundance of particular skin or stool bacterial species were associated with the MVA-Nab response. Abundant *Prevotella* in the skin at baseline was positively correlated with MVA-Nab response. *Prevotella* is known to promote mucosal inflammation and to stimulate production of epithelial cell cytokines (49). *Prevotella* is also found in larger numbers in the skin of women aged 60-76 years than in that of women in their 20s and 30s and was enriched in all of the skin sites of the older group compared to the younger ones (50). In stool, we found that *Eubacterium* abundance at baseline was positively correlated with the MVA-Nab response. This family of bacteria is known to be associated with gut health (51–53), and several of its species are higher in centenarians than in either young or elderly adults (54). The potential impact of the gut microbiota on vaccine immunogenicity has been already investigated with systemic vaccines (55) and with oral vaccines including those of rotavirus (RVV), polio, and cholera, mainly in infants/children living in low-income countries (56, 57). For example, bacterial species related to *Streptococcus bovis* species were more abundant before vaccination in Ghanaian vaccine-responders than non-responders and were positively associated with RVV efficacy, whereas *Bacteroides* and *Prevotella* species were more common in the microbiome of nonresponders and correlated with a lack of RVV response (58). In Bangladeshi infants, the pre-vaccination presence of *Bifidobacterium* was positively associated with some adaptive immunological responses, such as CD4^+^ and CD8^+^ T-cell proliferative responses to BCG and tetanus toxoid vaccinations as well as specific IgG responses to tetanus toxoid and hepatitis B vaccines, whereas high levels of enteric pathogens such as Enterobacteriales and Pseudomonadales were associated with neutrophilia and poorer vaccine responses (55).

Secondly, we examined the pre-vaccination host blood genes that were correlated with MVA-Nab intensity. We then investigated microbiota abundance to decipher a minimal gene signature predictive of MVA-Nab responsiveness. Interestingly, within this signature we find BLK, IGLV8-61 and EBF1 involved in B cell development, proliferation and differentiation and in the positive regulation of transcription in B cells and B cell receptor signaling. The BLK gene belongs to the family of protein tyrosine kinases src, and the B cells activation induces BLK gene product phosphorylation playing a key role in transmitting signals through surface immunoglobulins which supports the pro-B to pre-B transition and the signaling for growth arrest and apoptosis downstream of B-cell receptor (59). BLK also plays a role in the development, differentiation, and activation of B cells and in the intracellular signaling pathway. BLK is detected in pro-B cells and persists in mature B cells but is absent in plasma cells. Triple protein tyrosine kinase (SFK)-deficient mice — BLK, LYN, and FYN — have impaired NFkB signaling and B cell development (60). EBF1, an early B cell factor 1, is one of the transcription factors essential for orchestrating the development of the B cell line. Heterozygosity of EBF1 results in the deregulation of at least eight transcription factors involved in lymphopoiesis and the deregulation of key proteins that play a crucial role in the survival, development, and differentiation of pro-B cells (61). IGLV8 (variable domain) is a glycoprotein produced by B lymphocytes; its binding of a specific antigen triggers the clonal expansion and differentiation of B lymphocytes into immunoglobulin-secreting plasma cells. The link between microbiota and host blood transcriptome has also been studied previously by Nakaya et al., who showed that TLR5 expression in blood 3 days after influenza vaccination was correlated with antibody response 28 days later (28). This correlation was significantly lower in TLR5-deficient mice immunized with TIV compared to wild-type mice. As influenza vaccine does not stimulate TLR5 directly, however, Oh et al. demonstrated with germ-free or antibiotic-treatment that the commensal bacteria were the source of the TLR5 ligands responsible for enhancing immune response to TIV (7). It should be noted that in our study the three genes were negatively correlated with MVA-Nab response and microbial diversity of both skin and stool samples but also with the abundance of the *Prevotella* family in skin and the *Eubacterium* family in stool. The logistic regression based on the expression of these three genes and *Prevotella* and *Eubacterium* abundance for, respectively, skin and stool, highlights the predictive power of this signature for the MVA-Nab immune responses. These results propose that an advanced differentiation state of B lymphocytes before vaccination, potentially represented by a high expression of these three genes and associated with low genus abundance and diversity, might be associated with poor MVA-Nab response.

## Data Availability Statement

The datasets presented in this study can be found in online repositories. The names of the repository/repositories and accession number(s) can be found below: https://www.ebi.ac.uk/arrayexpress/, E-MTAB-9642; https://www.ncbi.nlm.nih.gov/, PRJNA691892; and https://www.ncbi.nlm.nih.gov/, SAMN17307480 to SAMN17307549.

## Ethics Statement

The studies involving human participants was conducted in accordance with the Declaration of Helsinki and the International Conference on Harmonization Good Clinical Practice guidelines and approved by the relevant regulatory and independent ethics committees. Each participant provided written informed consent before study entry. The study was registered and approved by the Peru regulatory authorities (IMPACTA IRB 0037-2014-CE; Peru NIH 396-2014-OG-OGITT-OPE/INS). The patients/participants provided their written informed consent to participate in this study.

## Author Contributions

Conceptualization (BC and CB). Methodology (BC, CB, JS, and RP). Funding (BC, CB, JS, and RP). Acquisition and validation (EG and YG). Formal analysis (EG, YG, and BC). Investigation (EG, YG, BC, and RP). Supervision (BC, CB, JS, JL, and RP). Resources (BC, CB, JS, JL, and RP). Data curation (EG and YG) - Writing (EG, YG, and BC) - original draft preparation. Writing (EG and YG) – Review Editing (all authors). Visualization (EG and YG). Project administration (BC, CB, JS, and RP). All authors contributed to the article and approved the submitted version.

## Funding

This project has received funding from the European Union’s Horizon 2020 research and innovation programme under grant agreement No. 681137, and support by the Fondation Dormeur, Vaduz, (Liechtenstein). RP and CB were partly funded from the European Union’s Horizon 2020 Research and Innovation programme under the grant agreement no 847943 (MISTRAL).

## Conflict of Interest

CB is co-founder, shareholder and employee of Aelix Therapeutics, outside of this work.

The remaining authors declare that the research was conducted in the absence of any commercial or financial relationships that could be construed as a potential conflict of interest.
