# Supplementary material for: Host Transcriptome and Microbiota Signatures Prior to Immunization Profile Vaccine Humoral Responsiveness
Source: Front Immunol. 2021 May 10;12:657162. doi: 10.3389/fimmu.2021.657162 (PMC8141841; doi:10.3389/fimmu.2021.657162)
Supplement: Supplementary file 1 [file Image_1.pdf]

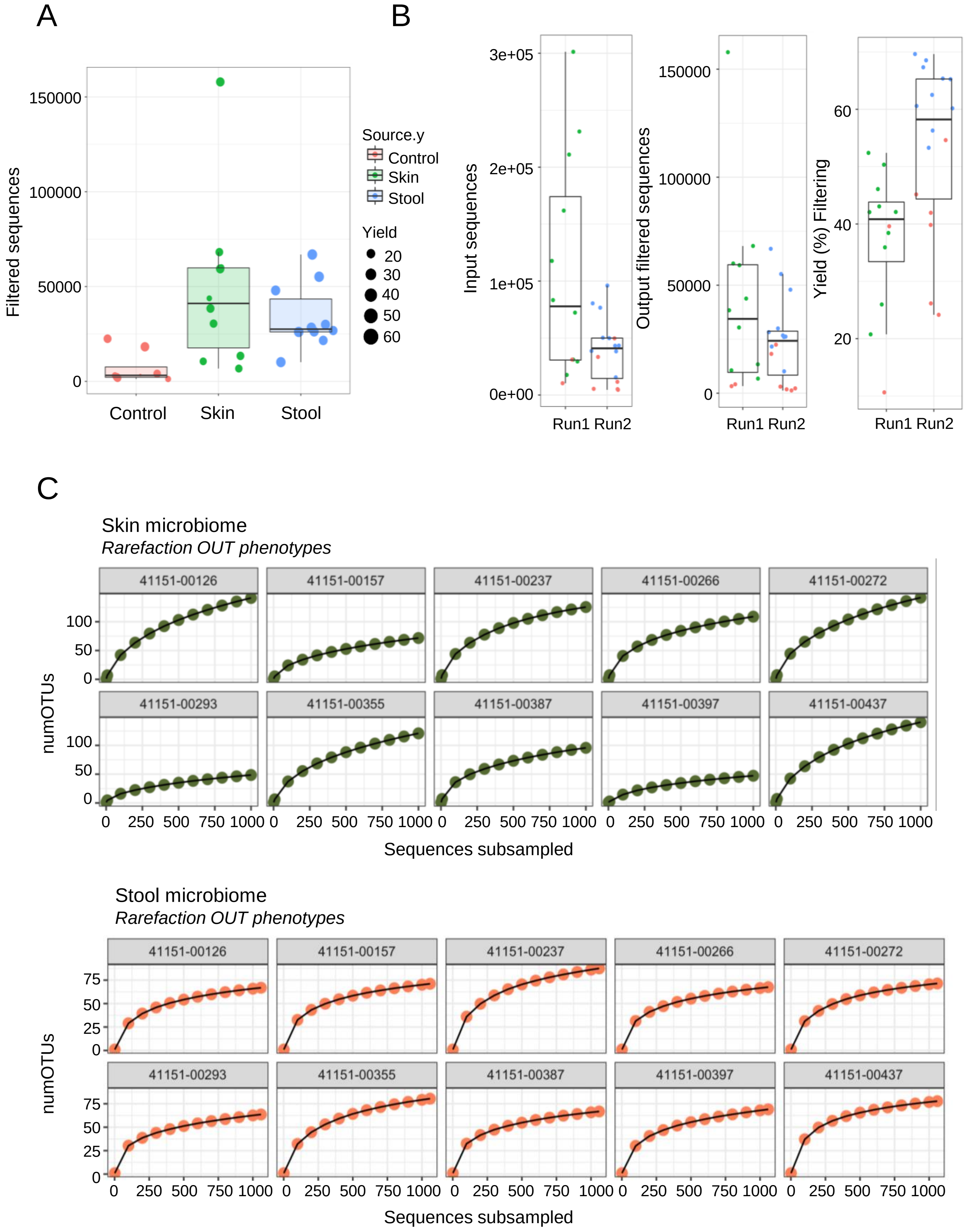
A
Filtered sequences
150000
100000
50000
0
Control
Skin
Stool
Source.y
Control
Skin
Stool
Yield
20
30
40
50
60
B
Input sequences
3e+05
2e+05
1e+05
0e+00
Run1 Run2
Output filtered sequences
150000
100000
50000
0
Run1 Run2
Yield (%) Filtering
60
40
20
Run1 Run2
C
Skin microbiome
Rarefaction OUT phenotypes
41151-00126
41151-00157
41151-00237
41151-00266
41151-00272
41151-00293
41151-00355
41151-00387
41151-00397
41151-00437
numOTUs
100
50
0
0 250 500 750 1000
Sequences subsampled
Stool microbiome
Rarefaction OUT phenotypes
41151-00126
41151-00157
41151-00237
41151-00266
41151-00272
41151-00293
41151-00355
41151-00387
41151-00397
41151-00437
numOTUs
75
50
25
0
0 250 500 750 1000
Sequences subsampled

Supplemental Figure 1. Sequences quality control for microbiota analyses (A) Total number of filtered sequences per sample in control, skin and stool samples. **(B)** From left to right: Distribution of the number of total raw sequences (Input sequences), filtered sequences (Output filtered sequences), and filtering yield values per each type of sample, grouped by the two independent sequencing batches (Run1 and Run2). **(C)** Rarefaction curves for skin and stool samples. Panel identifiers correspond to different individuals.
